# Gastrostomy Placement and Management in Children: A Single-Center Experience

**DOI:** 10.3390/nu11071555

**Published:** 2019-07-10

**Authors:** Grazia Di Leo, Paola Pascolo, Kamar Hamadeh, Andrea Trombetta, Sergio Ghirardo, Jurgen Schleef, Egidio Barbi, Daniela Codrich

**Affiliations:** 1 Pediatric Gastroenterology, Endoscopy and Nutrition Unit, Institute for Maternal and Child Health, IRCCS Burlo Garofolo, via dell’Istria 65/1, 34137 Trieste, Italy; 2 Pediatric Clinic, Maternal and Child Department, San Polo Hospital, Azienda Bassa Friulana-Isontina n.2 (AAS2), via Galvani 1, 34074 Monfalcone (GO), Italy; 3 Department of Medical, Surgical and Health Sciences, University of Trieste, 34149 Trieste, Italy; 4 Department of Paediatric Surgery, Institute for Maternal and Child Health, IRCCS Burlo Garofolo, via dell’Istria 65/1, 34137 Trieste, Italy; 5 Pediatric Department, Institute for Maternal and Child Health IRCCS, Burlo Garofolo of Trieste, via dell’Istria 65/1, 34137 Trieste, Italy

**Keywords:** percutaneous gastrostomy, children, neuromuscular disease

## Abstract

BACKGROUND: To prevent malnutrition and food aspiration in children with chronic neuromuscular problems, enteral nutrition provided by gastrostomy is recommended. Long-term follow-up data about surgical and medical complications of PEG are available, but few papers have addressed all of the issues in the same series. METHODS: This retrospective study enrolled patients under 18 years who had a gastrostomy tube placed at our institution between 2003 and 2017. The aim is to evaluate outcomes after gastrostomy placement, focusing both on surgical complications (early and late), and its effect on their nutritional status, on the prevention of pulmonary infections, and their parents’ opinion. RESULTS: Eighty-four gastrostomies were placed in total (35 F; 49 M). Seventy-seven patients had a severe neurocognitive impairment (GMFCS 5). The principal indication for gastrostomy was severe dysphagia (53.3%). No gastrostomy-related death was observed. Early surgical complications were observed in five of 84 (5,9%) patients; late complications were observed in 15 of 84 (17.8%) patients. Twenty-two patients were diagnosed with subsequent gastroesophageal reflux; five patients developed dumping syndrome (6%). Complete medical follow-up data were available for 45 patients. A progressive improvement of nutritional status was observed in 29 patients, and 11 maintained the same percentile; the occurrence of respiratory infections and need for hospitalization decreased. In 90% of cases, parents were fully satisfied with the g-tube. CONCLUSION: This study confirms the positive nutritional outcomes of gastrostomy-tube with an associated small risk of surgical complications and a reduction in the number of respiratory infections, with most parents scoring their experience as positive.

## 1. Introduction

Neuromuscular disorders (NMDs) encompass heterogeneous conditions, including motor neuron diseases, neuropathies, disorders of the neuromuscular junction, and myopathies. The most common motor disability in childhood is represented by cerebral palsy (CP) [1], defined as a group of the motor, cognitive, and perceptive impairments secondary to a non-progressive defect or lesion of the developing brain [2]. Immobility and muscular weakness are underlying features of all these disorders, which predispose the sufferers to nutritional and infective complications [3].

In particular, reduced nutritional intake due to oropharyngeal dysfunction [4], temporo-mandibular joint contractions, and sensory impairment leads to malnutrition in most of the cases, with severe motor dysfunction [4,5].

Furthermore, the impaired ability to coordinate swallowing with ventilation predisposes sufferers to food aspiration. Recurrent aspiration pneumonia episodes lead to progressive lung parenchymal damage [6], worsened by compromised airway defense mechanisms, and may cause death by reflux aspiration.

To prevent malnutrition and food aspiration in a functional gastrointestinal tract, enteral nutrition (EN) support is recommended [7]. In particular, gastrostomy can provide long-term enteral nutrition (EN) in chronically ill children [8] with neuromuscular problems [9].

While some studies have evaluated the efficacy and side effects of gastrostomy from surgical or medical perspectives, still limited data are available on both aspects in the same series and on a long-term follow-up [10,11,12]. To our knowledge, no data are available about the incidence of a serious complication such as dumping syndrome, and limited data are available about the eventual need for a fundoplication procedure in a cohort of children all previously evaluated before gastrostomy positioning for gastro-esophageal reflux, as in this study.

The aim of this study is to evaluate the long-term follow-up of gastrostomy in children with neuromuscular disorders (NMDs), in particular in children with cerebral palsy (CP), focusing both on surgical complications and on their effects on the improvement of nutritional status and prevention of pulmonary infections.

## 2. Materials and Methods

### 2.1. Patients and Methods

This is a retrospective study conducted in the Institute for Maternal and Child Health Burlo Garofolo of Trieste, Italy.

We included retrospectively all patients who had a gastrostomy-tube placed at our Institution between January 2003 and December 2017. 

The primary outcome was the percentage of patients that suffered one or more complications. The secondary outcome was the effect of gastrostomy in terms of nutritional status, number and severity of respiratory infections, type, and duration of meals. We finally evaluated patients’ and families’ opinions about the gastrostomy.

All of the included children’s parents provided written, informed consent to access medical records for research purposes.

Data were collected from the clinical records of Gastroenterology and Clinic Nutrition Unit and Pediatric Surgery and Urology Unit. 

For each enrolled patient, the following variables were collected: gender, date of birth, and if present, date of death, the degree of intellectual disability if present, and the diagnosis.

Four main indications for gastrostomy were identified: malnutrition, severe dysphagia, risk of inhalation, and intractable food aversion. Malnutrition was defined as a weight inferior to the 10th percentile on disease-specific centiles when available [13], or as the impossibility to gain or maintain weight. Severe dysphagia was defined by barium swallow x-ray with concomitant inability to meet 60% to 80% of individual requirements or total feeding, or time longer than six hours per day [14]. Risk of inhalation was defined as the occurrence of pneumonia related to reflux aspiration. Intractable food aversion was defined as a persistent food refusal reflected in significant failure to gain weight or significant weight loss (>1 month), in absence of obvious organic disease, with pathological feeding or anticipatory gagging [15].

We registered the type of placement as endoscopic or surgical (laparoscopic or open). The procedure was surgically performed when associated with another surgery (i.e., fundoplication) or if the transillumination was judged inadequate and a laparo-assisted procedure was considered safer. All the endoscopic placements were performed under general anesthesia in the operating room with endotracheal intubation by a team consisting of an attending pediatric gastroenterologist, a gastroenterology nurse, a pediatric surgeon, and a pediatric anesthesiologist. All patients received a preoperative dose of antibiotics (generally cephalosporin) immediately before PEG followed by two other subsequent doses.

We recorded the type of surgery and intra-operative or post-operative complications distinguishing them in early (occurred during the first 30 days after gastrostomy placement) and late (occurred 30 days or more after PEG placement) phases. We considered major complications, such as gastric bleeding, aspiration pneumonia, massive pneumoperitoneum, gastrocolic fistula, cutaneous necrosis, or need for surgical closure of the gastro-cutaneous fistula after g-tube removal and dumping syndrome. Minor complications were local problems, such as erythema, granuloma, local infections, leakage, and dislocation. 

Presence of gastro-esophageal reflux disease was routinely evaluated before and after g-tube placement. We also recorded the occurrence of other medical problems (such as dumping syndrome) after gastrostomy and the need for a subsequent fundoplication. 

We registered data of patients three times: at the placement of the gastrostomy (T0); at the nutritional evaluation after one year since the gastrostomy (T1); and at the last nutritional follow-up visit available (T2).

At each time, nutritional status and lung infections were recorded. Nutritional data include weight and weight percentile. For children aged 2–18 years with neurocognitive impairment, we used specific growth charts for the weight (weight for age percentile) related to motor function using the Gross Motor Function Classification System (GMFCS level 5) [13]. For those between 0 and 2 years, the World Health Organization (WHO) growth charts standard were used (weight for age) [16]. The number and the severity of respiratory infections were registered and we distinguished four categories: (A) Children without significant lower respiratory infections requiring antibiotic treatment; (B) children who presented from 1 to 4 episodes of mild respiratory infections requiring antibiotics; (C) more than 5 episodes of respiratory infections requiring antibiotics; (D) children with severe respiratory infections that required hospitalization.

At T2 we registered dietetic information, in particular, type (pureed food or high-calorie formula) and volume of food, and time spent feeding. 

At T2 parents were asked to judge their experience with the gastrostomy procedure by a single Number Rating Scale (NRS) from 0 to 10. A score below 7 was arbitrarily considered as a negative judgment. Moreover, we queried parents about their personal comments on g-tube.

An attending surgeon recorded surgical complications, while a gastroenterologist collected medical and nutritional information. Exclusion criteria: children whose parents refused consensus for data use and children undergoing jejunal tube positioning were not included in the study.

### 2.2. Statistical Analysis

For the primary outcome, we reported the absolute number of complications and the percentage of patients that presented one or more of these complications on the total number of patients. The absolute number of minor complications was also reported, as well as the percentage of patients that presented one or more of these; the same applied for each discrete variable and dichotomic ones. We reported the absolute number and the percentage of patients with malnutrition and pulmonary infections at each reevaluation T0, T1, and T2, and evaluated the impact of the intervention using the Wilcoxon signed-ranks test to compare T0 and T2 in terms of class of pulmonary infections and decile of weight.

## 3. Results

Between 2003 and 2017, 84 gastrostomies were placed in our Institution. Based on medical records, a register of patients with gastrostomy was created.

Of 84 patients, 35 were female (41.6%) and 49 were male (58.3%). The median age at the time of gastrostomy was 4.5 years, the mean was 7.1 years, the range was 1 month to 29 years, the first quartile was 1 year, and the third quartile was 13 years. The median age at the last follow-up was 12.5 years, the mean was 13.2 years, the range was 13 months to 29 years, the first quartile was 9 years, and the third quartile was 35 years.

Seventy-seven patients have severe neurocognitive impairment (GMFCS 5) (see Table 1). The other seven (8.3%) are children with normal neurocognitive development (six children with operated esophageal atresia, and one child with spinal muscular atrophy). The principal indication for gastrostomy is severe dysphagia (53.3%); frequencies for each indication are shown in Table 1. 

An endoscopic gastrostomy was performed in 63 patients (75% of patients), with a standard “pull” technique in 59 cases and a push technique for the other 4 patients; a surgical approach was used for the remaining 21 patients (25% of patients). Out of this 21 who underwent a surgical open or laparoscopic gastrostomy, sixteen (76.2%) had a concurrent fundoplication during the same procedure. Twenty-one children (23.8% of the total, 33.3% of the remaining) needed a subsequent fundoplication, while 5 children underwent fundoplication before gastrostomy; one of them needed a surgical revision of the fundoplication after the gastrostomy.

Five patients (5.9%) presented early complications (intraoperative), of which three were considered major complications (intestinal perforation, liver injury, massive pneumoperitoneum requiring exploration for suspected perforation) and two were minor (omental leakage from surgical wound, mild and self-limiting pneumoperitoneum in one case). There was no gastrostomy-related death. Late complications (postoperative) were observed in 15 of 84 (17.8%) patients (see Table 2). 

Twenty-two patients out of 84 underwent fundoplication at the same time of g-tube placement. Of the remaining 62, twenty-two (35.5%) developed gastroesophageal reflux (GERD) after g-tube placement. Of them, 19 underwent a subsequent fundoplication. One case underwent a reintervention on the previous fundoplication. Another patient that underwent fundoplication with g-tube placement developed a subsequent GERD that was not responsive to medical treatment. 

Five patients out of 84 developed a dumping syndrome (5.95%) with repeated episodes of severe symptomatic hypoglycemia.

During follow-up, 14 children died. No death was related to gastrostomy placement. In six children, death was attributed to reflux inhalation; all of them had a fundoplication and were fed by g-tube exclusively. Two patients were reported to have died from acute severe dehydration episodes, two from untreatable epileptic status, four from heart failure, and two due to respiratory failure. 

**Figure 1:**
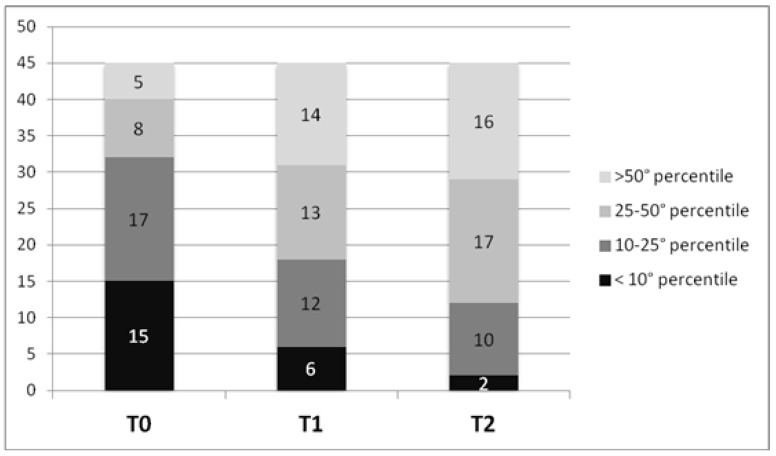
At each time (T0, T1, T2) patients are subdivided in groups based on the weight percentile.

Out of 84 patients, only 45 were considered for the nutritional analysis, due to incomplete nutritional data at follow-up in the others. The mean weight gain was + 5.15 kg at T1 (standard deviation SD 4.42), and +12.6 kg at T2 (SD 9.7). Based on growth charts (for age and motor function), of 45 patients, 15 children 33.3% were malnourished (weight percentile < 10°) at time of gastrostomy placement. As shown in Figure 1, the number of malnourished children decreased progressively at T1 and T2, and increased the number of children with a weight above the 25° percentile. The increase in decile of weight between T0 and T2 is highly statistically significant (*p* < 0.0001 using Wilcoxon signed-ranks test).

Two children did not show any improvement: one was a boy with congenital cardiomyopathy in Vici’s syndrome who died 6 months after gastrostomy placement; the second one underwent gastrostomy placement at the age of 16 months, when he weighed 13.5 kg, and with a total weight gain of +4.6 Kg in 3.8 years. 

The leading indications for g-tube for children with weight percentile over the 25° percentile were dysphagia and risk of inhalation. All of them maintained or improved their nutritional status.

Immediately after g-tube placement, hypercaloric formula (1kcal/mL) was the main food in 28 out of 45 patients (62.2%), while at the last follow-up, 24 patients used it out of 32 patients that were still alimented through gastrostomy (75%). Two patients exclusively received self-prepared food, while 6 received a combination of self-prepared foods and hypercaloric formula; every patient was fed through a gastrostomy.

The reported average time spent in feeding the children was 2.8 h (SD +/− 1.5) a day, comprising time spent on orally tasting food flavor in three patients.

At the last follow-up of this subset of patients, 37 patients were still alive. Five patients out of 37 (13.5%) removed the g-tube: in four patients this was due to a satisfying oral intake, and for one patient due to the occurrence of a cutaneous-gastric fistula. 

At T0, the presence of respiratory infection was considered determinant for the decision to perform gastrostomy for 12 patients, with 10 of them requiring repeated hospitalization and 5 of them having undergone fundoplication previously.

At T1, respiratory infections were reported as rare or not relevant in 42 patients, and only two patients required hospitalization at T1 for such a condition. The occurrence of respiratory infections is displayed in Table 3.

The reduction of the class of respiratory infection was statistically significant (0.00037 using Wilcoxon signed-ranks test).

Thirty-six parents out of 45 answered that the average NRS score was 8 (SD 1.5). Of these, five reported that they were not satisfied (score < 7; negative judgment), one because of the leakage of formula and gastric contents, two other families reported that the button did not hold well, and two others because of frequent dislocation.

## 4. Discussion

This study shows that g-tube positioning is effective in improving nutritional status and reducing lung infections in children with cerebral palsy or dysphagia related to other conditions, with a low rate of major complications and an average of high parental satisfaction.

Our population is comparable to other similar case series for main characteristics, such as gender, diagnosis, indication for gastrostomy, and age of g-placement. 

According to the literature, we observed high mortality during the study period (sixteen patients, 19% of patients’ cases), however not linked in any case to the insertion of the gastrostomy. In all cases, the causes of death are related to the underlying pathology and its complications, reflecting that many children who need gastrostomy have severe underlying disease with reduced life expectancies. 

As far as surgical complications are concerned, the complication rate in literature is various. Comparing with Fortunato and colleagues [17], who reported the largest series of such patients (760 patients), our population has a comparable rate of early complications (5.9% in our population, versus 4%) and no death related to the surgical procedure was reported. Despite a longer follow-up in our experience, the rate of late complications is similar (17.8% versus 20%). 

Comparing with more recent literature on 326 patients who underwent PEG positioning (Lalanne 2014) [18], the rate of late complications is lower (17.8% versus 56%), with a comparable duration of follow-up.

Despite the lack of recommendations in the literature [19,20], in our institute, we routinely evaluate the presence of GERD and its severity through esophageal pH-monitoring before and after gastrostomy and perform fundoplication only if the reflux is symptomatic. Altogether, GERD involved half of the patients, a quarter of which were identified before placement and a quarter after placement, meaning that a routine fundoplication would result in 50% unneeded surgical procedures.

Due to the retrospective nature of the study, we are not able to define if GERD is a g-tube complication or a complication related to the underlying disease [21].

Nevertheless, our results seem to support the need for appropriate evaluation and re-evaluation for GERD and fundoplication case-by-case due to the relatively high incidence of GERD after gastrostomy.

We reported a 6% incidence of dumping syndrome. In the literature, there are small case series of dumping syndrome following Nissen fundoplication [22]. To the best of our knowledge, we could not find references to its prevalence in children who performed a gastrostomy. Dumping syndrome with hypoglycemia is a severe complication, which may be difficult to recognize and misdiagnosed resulting in worsening of epilepsy when present [22]. When suspected and recognized, it can be easily managed, and our results suggest that it should be considered as a somewhat common complication of enteral nutrition by a g-tube.

As far as nutrition status is concerned, in accord with the literature [18,19], we also observed a satisfying improvement of nutritional status. 

According to the literature, our results confirm that the gastrostomy placement (either endoscopic either surgical) does not increase the risk for aspiration pneumonia. In addition to literature, we found a positive impact on respiratory infection rate leading to a minor need for hospitalization and improvement of quality of life. 

This study has some limitations—it is retrospective, and we reported a high number of patients with incomplete data or lost at the gastroenterological follow-up. Furthermore, the evaluation of nutritional status is limited by the lack of triceps skinfold evaluation. 

The main point of strength of our study is that this is one of the few studies considering all of the different issues, such as surgical complications, nutritional status, infection rate, and parent satisfaction in a homogenous population at a reasonable length of follow-up.

## 5. Conclusions

This study confirms the positive nutritional outcomes of PEG with a small risk of early and late surgical complications and a reduction in the number of respiratory infections, with parents scoring their experience as positive.

We hope that a more detailed knowledge of gastrostomy outcome can help pediatricians and families in the decision-making process of g-tube placement.

## Figures and Tables

**Table 1:** Indication for g-tube placement related to patients’ diagnosis.

Diagnosis Number (%)		Indications for Gastrostomy-Tube			
		Malnutrition 18 (21.4%)	Risk of Inhalation 9 (10.7%)	Severe Dysphagia 51 (60.7%)	Food Aversion 6 (7.1%)
Cerebral palsy	56 (66.7%)	11	8	37	--
Genetic disorder	13 (15.5%)	5	--	7	1
Metabolic or other	9 (10.7%)	2	1	5	1
Esophageal atresia	6 (7.1%)	--	--	2	4

**Table 2:** Late complications of gastrostomy tube placement.

Late Complications	
Major	Minor
surgical revision 3 (3.6%) occlusion 2 (2.4%) buried bumper syndrome 2 (2.4%) dumping Syndrome 5 (5.9%)	dislocation 10 (11.9%) granuloma or skin infection 7 (8.3%)

**Table 3:** Patients subdivided by number and severity of respiratory infections at T0 and T2.

	T0	T2
Patients with infection related hospitalization (D)	10	1
Patients with more than 5 episodes of respiratory infection (C)	2	1
Patients with 1 to 4 episodes of mild respiratory infection (B)	16	16
Patients without significant respiratory infection (A)	17	27
